# Delivery Timing, Out‐Of‐Pocket Maternity Costs, and Postpartum Care Utilization: An Instrumental Variable Analysis Among Commercially Insured Women

**DOI:** 10.1111/1475-6773.70161

**Published:** 2026-09-02

**Authors:** Rebecca A. Gourevitch, Jessica L. Cohen, Tara Shakley, Sung Min Park, Mary Beth Landrum, Meredith B. Rosenthal, Mark W. Friedberg, Anna D. Sinaiko

**Affiliations:** ^1^ Department of Health Policy and Management University of Maryland College Park Maryland USA; ^2^ Department of Global Health and Population Harvard T.H. Chan School of Public Health Boston Massachusetts USA; ^3^ Blue Cross Blue Shield of Massachusetts Boston Massachusetts USA; ^4^ Department of Health Care Policy Harvard Medical School Boston Massachusetts USA; ^5^ Department of Health Policy and Management Harvard T.H. Chan School of Public Health Boston Massachusetts USA

## Abstract

**Objective:**

To examine whether out‐of‐pocket costs during pregnancy and delivery affect use of postpartum care.

**Study Setting and Design:**

Because health insurance deductibles and limits reset annually, the timing of childbirth within that year quasi‐randomly assigns people to different levels of cost‐sharing during pregnancy+delivery versus postpartum. We use a novel instrumental variable approach that leverages this variation to analyze whether higher maternity spending due to delivering early in the plan year affects postpartum care utilization. The exposure is maternity out‐of‐pocket spending, and the instrument is whether the delivery was in the first three versus last 3 months of the enrollee's health plan year; the primary outcome is use of any outpatient care postpartum. We analyze maternity episodes among Blue Cross Blue Shield of Massachusetts enrollees who gave birth, 2019–2023.

**Data Sources and Analytic Sample:**

Commercial health insurance enrollment and claims data for individuals with continuous enrollment during pregnancy, delivery, and 3‐months postpartum (*N* = 51,337).

**Principal Findings:**

Out‐of‐pocket costs for those delivering at the start versus the end of their health plan year were, on average, 21% higher for pregnancy+delivery care and 58% lower for postpartum care. A $100 increase in pregnancy+delivery out‐of‐pocket spending led to a 0.53 percentage point (95% CI [0.28, 0.79]) increase in use of any outpatient postpartum care (sample mean: 82.5%). Higher pregnancy+delivery out‐of‐pocket costs also led to significant increases in the number of outpatient contact days, visits for preventive/well care, visits for mental health, and other visits.

**Conclusions:**

Higher pregnancy+delivery out‐of‐pocket costs due to delivering early in the plan year corresponded to lower postpartum out‐of‐pocket costs and led to modest increases in postpartum care. This suggests that lower postpartum cost‐sharing may increase postpartum care use. Policies that lower those costs may be effective in increasing use of postpartum care.

## Callout Box

- What is known on this topic.
  ○ Maternity care has high out‐of‐pocket costs for commercial insurance enrollees and is associated with problems paying medical bills. The impact of these costs on postpartum care use is unknown.
  ○ Patients with large bills may limit future utilization; patients use more care when out‐of‐pocket costs become lower (e.g., after meeting their deductible). Both mechanisms could affect postpartum care use.
  ○ Our study examines how the level of pregnancy, delivery, and postpartum out‐of‐pocket costs influences patients' use of care during the 3 months after childbirth.
- What this study found.
  ○ The timing of a childbirth within a health plan year strongly predicts the level of out‐of‐pocket spending for pregnancy, delivery, and postpartum care.
  ○ Higher out‐of‐pocket spending during pregnancy and delivery, which on average corresponds to lower cost‐sharing during the postpartum period, led to modest increases in use of postpartum outpatient care.
  ○ Reducing postpartum cost‐sharing in commercial insurance is a potential strategy for increasing use of postpartum care.

## 1 Introduction

Maternity care carries high out‐of‐pocket costs—$3133 on average—for people with commercial insurance, who make up half of all childbirths in the United States [1]. Although the Affordable Care Act requires prenatal visits and some prenatal services be exempt from cost‐sharing, important prenatal services (e.g., blood tests, ultrasonographic examination, and fetal surveillance for common morbidities) and delivery hospitalizations are not exempt and may be subject to cost‐sharing. Childbirth increases the likelihood of having catastrophic health care expenditures and problems paying medical bills, particularly among patients with pregnancy complications or who have low incomes [2, 3, 4, 5]. Maternity costs fall disproportionately on Black and Hispanic birthing people, and represent a higher share of their household income [6]. Nearly half of commercially‐insured birthing people were still paying off childbirth‐related medical debt 1 year postpartum [7]. These costs coincide with many additional expenses due to having a baby, and, for many, unpaid time off of work. To improve affordability, current state and federal policy proposals are considering eliminating cost‐sharing for pregnancy, delivery, and/or postpartum care [8, 9].

High costs of pregnancy and delivery care could also impact healthcare utilization following childbirth, through multiple mechanisms. First, having significant medical expenses will decrease the patient's available budget for spending on other things. This tighter budget (an income effect, in economic terms) can decrease consumption of all goods and services, including healthcare. Second, receiving a high medical bill for delivery may also specifically deter use of postpartum health care services out of a concern of additional medical bills; a recent study found evidence of this “bill shock” effect among commercially insured patients [10]. Another factor influencing healthcare utilization in the postpartum period is patient cost‐sharing. Postpartum out‐of‐pocket costs could deter postpartum care utilization—a “price effect”—and these prices will vary during a plan‐year in part based on cumulative health spending in that year.

Prior work in US settings has examined how the demand for healthcare responds to prices. Even small out‐of‐pocket costs reduce patient's use of high‐value health care services relative to patients with no cost‐sharing [11, 12, 13]. In addition, there is heterogeneity in price response by type of health care, with more response (i.e., demand is more price elastic) for outpatient care and less for inpatient care [13, 14]. Patients respond to the nonlinear cost‐sharing structure of insurance contracts with deductibles in ways consistent with myopia—or only considering short‐term prices [15, 16, 17]. In practice, this means that people tend to use less care before they have met their deductible, and then increase utilization after they have met their deductible and they face lower costs at the point of care. Most existing US‐based studies lack the data needed to examine the impacts of price and income effects together. In one analysis, the RAND Health Insurance Experiment found that income plays a smaller role compared to cost‐sharing (price) in determining healthcare demand [11].

There is little evidence on the impact of cost‐sharing for pregnancy, delivery, and postpartum care on use of postpartum care. Early and frequent outpatient postpartum visits are recommended to improve postpartum outcomes (most pregnancy‐related deaths and a substantial share of severe maternal morbidities occur after delivery) [18, 19], but many patients do not receive care [20, 21]. Increasing access and use of postpartum care has been a national policy priority that has ushered in policy changes including extending Medicaid coverage beyond 60 days postpartum. However, there may be barriers to postpartum care in the commercially insured population that are not present in Medicaid, because Medicaid prohibits cost‐sharing for maternity‐related services while commercial plans do not. For those with commercial insurance, the routine postpartum follow‐up visit with the obstetrician is exempt from cost‐sharing per the Affordable Care Act, but other services provided during the postpartum period are subject to the cost‐sharing requirements in the health plan.

**FIGURE 1:**
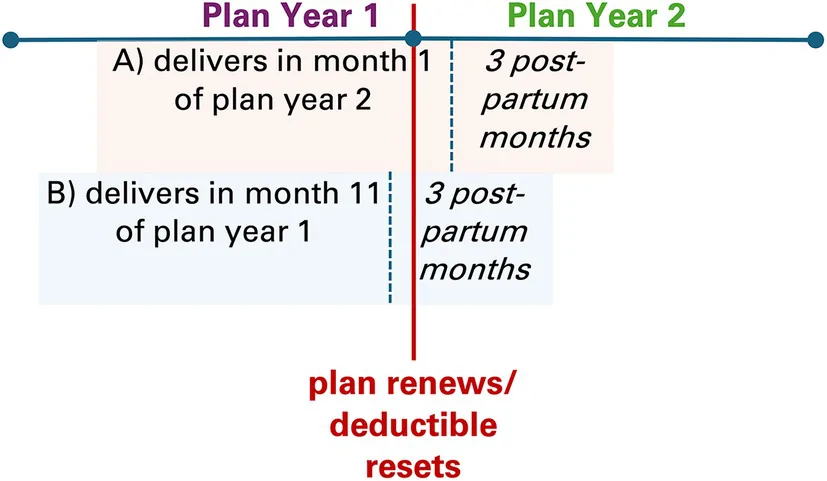
Timing of pregnancy, delivery, and postpartum periods across health plan years for a person delivering early (person A) vs late (person B) in the health plan year.

This paper examines the relationship between out‐of‐pocket costs during pregnancy, delivery, and the postpartum period, and how these costs impact use of postpartum care. Health insurance benefits are tied to a 1‐year period, after which deductibles reset. We exploit the fact that the timing of childbirth within a health plan year impacts the magnitude of cost‐sharing for the pregnancy and delivery (the income effect) and for postpartum care (the price effect). Figure 1 presents a simplified depiction of these dynamics for two people in the same health plan. Childbirth can happen at any point during the health plan year, and over the course of this high‐cost episode, most people reach their full deductible. However, those who deliver early in their plan year, like birthing person A in Figure 1, have a maternity episode that spans two health plan years; they therefore face and satisfy two deductibles over the pregnancy and delivery. Because they satisfied their Plan Year 2 deductible during delivery, they face lower, postdeductible cost‐sharing in the early postpartum period. In contrast, people delivering at the end of a health plan year, like B in Figure 1, pay only one deductible for their pregnancy and delivery care, and face their Plan Year 2 deductible during their early postpartum period. Person B's out‐of‐pocket costs for pregnancy and delivery are lower or equal to Person A's out‐of‐pocket costs, and Person B has the same or higher out‐of‐pocket costs as Person A for postpartum care because their deductible has reset.

The timing of a childbirth episode within a health plan year quasi‐randomly assigns people to different levels of cost‐sharing for pregnancy+delivery and postpartum care. We use a novel instrumental variable approach that leverages this variation to estimate whether higher pregnancy+delivery spending due to delivering early in the plan year decreases (i.e., the income effect dominates) or increases (i.e., the price effect dominates) use of postpartum care. While papers have used the health insurance calendar reset as a source of exogenous variation in cost‐sharing [16, 17, 22], to our knowledge, there has not been an analysis that uses different plan reset dates and variation in timing of health events in an instrumental variable strategy to estimate how cost‐sharing effects demand for care in the United States. We hypothesized that, because the higher cost‐sharing from a second deductible during delivery is a large amount, the income effect would dominate. We also examine these effects in the subgroup of people with a high‐deductible health plan, who, on average, face higher pregnancy+delivery costs and therefore would have experienced a larger income effect.

## 2 Methods

### 2.1 Data and Study Sample

We conducted a retrospective, observational study using enrollment and claims data from 2019 through 2023 from Blue Cross Blue Shield of Massachusetts (BCBSMA), a large health insurance company covering approximately 3 million lives annually. The data include health plan benefit design details including the deductible level, product type (Health Maintenance Organization (HMO) vs. Preferred Provider Organization (PPO)), and the month in which the health plan‐year begins. This improves upon existing analyses that examined variation in out‐of‐pocket spending with timing of delivery, which assumed January start dates for all health plans [23].

Our sample includes all deliveries for which the birthing person was continuously enrolled in any BCBSMA plan through their pregnancy, delivery, and 3 months postpartum. This continuous enrollment requirement ensures that we observe all billed healthcare utilization and outcomes during this period. We focus on the first three postpartum months as that is the period of greatest postpartum healthcare use and limits the sample lost due to the continuous enrollment requirement [24]. In addition, it more precisely captures the period where “bill shock” would be observed in response to receiving the delivery bill; there is a median of 22 days between the delivery and the time BCBSMA pays for the care episode (Appendix A), which other work has used as a marker for the earliest date that enrollees could receive their bill [10].

We use billing codes to identify delivery episodes (Appendix B). We identify the start of each pregnancy using the weeks of gestation indicated by the International Classification of Diseases (ICD‐10) gestational age code on the delivery episode [25]. We define the postpartum period as the 3 months following the date of discharge on the delivery claim. We exclude 198 individuals (0.19%) who are missing data on the health plan‐month in which the delivery occurred.

### 2.2 Outcome Measures

Our outcomes capture care utilization in the 3 months following delivery (“postpartum period,” see Appendix C for detailed definitions). We measure use of any outpatient care in two ways: as a binary measure indicating any outpatient visit, as well as a continuous measure of the number of days with an outpatient visit (“outpatient contact days”). Following prior literature, we measure three types of outpatient visits: visits for preventive care/well care/postpartum evaluation, visits for contraceptive management, and visits for other acute or chronic illnesses [26]. Each of these is operationalized as a dichotomous measure indicating whether the postpartum episode included any of the visit types. These measures may not capture the standard 6‐week postpartum visit, which is often unobservable in claims data because it is paid as part of the maternity care global fee billed at the time of delivery. We therefore may underestimate the amount of postpartum care used, but this measurement error should be uncorrelated with the instrument and therefore would not bias our results.

We include two measures of mental health care use. We construct a binary measure indicating an outpatient mental health visit for people with a billing code indicating use of outpatient mental health care services. We construct a binary measure for any mental health medication equal to 1 if the person filled a prescription for an antidepressant or anxiolytic; this outcome was only included for enrollees with pharmacy coverage through BCBSMA during the postpartum period (69%). Finally, we include a dichotomous measure indicating whether the birthing person had any emergency department (ED) visits during the postpartum episode.

### 2.3 Exposure Measures

Our exposure measure is total out‐of‐pocket spending during pregnancy and delivery. We define this as the sum of all deductible, coinsurance, and copayment amounts on all claims during the patient's pregnancy period and delivery episode.

We measure the impact of pregnancy+delivery out‐of‐pocket spending using an instrumental variable approach. The instrument is the birthing person's “plan‐month of delivery,” which is the number of months into the person's health plan year when the birth occurred. For example, if a birthing person enrolled or was renewed in their health plan for coverage effective in January, and they gave birth in January, their plan‐month of delivery would be equal to 1 (e.g., person A in Figure 1). If that person instead gave birth in November, their plan‐month of delivery would be equal to 11 (e.g., person B in Figure 1). A person whose health plan year reset in July and gave birth in November would have plan‐month of delivery of 4.

### 2.4 Maternity Episode Characteristics

We measure birthing people's demographic, health, and insurance characteristics that may influence the relationship between pregnancy+delivery out‐of‐pocket spending and postpartum care utilization. From health insurance enrollment records, we measure categorical age at delivery and state of residence. Out‐of‐pocket spending during the maternal episode has been shown to vary significantly by race and ethnicity, and we measure these characteristics using a combination of self‐reported data and an imputation approach described in prior work [6]. We capture two health plan design characteristics at the time of delivery: whether they were enrolled in an HMO or PPO (product type) and whether their health plan had a high deductible, defined as a deductible at or above $1500 (the IRS' 2023 high‐deductible level). We use a validated algorithm to capture obstetric comorbidities documented during pregnancy or delivery and group the sample into quartiles of obstetric risk [27]. We define delivery episode length of stay as the days between the admission and discharge dates on their delivery claims and group the sample into tertiles of length of stay. We also capture whether the delivery mode was vaginal or cesarean using billing codes (Appendix B).

### 2.5 Analytic Approach

To understand the relationship between out‐of‐pocket spending during pregnancy+delivery and use of postpartum care, we require an empirical approach that overcomes the confounders that drive both spending and utilization. While some of these confounders are observable (e.g., documented health risks, benefit design), others are not (e.g., provider practice patterns, underlying propensity to seek care). To address these endogeneity concerns, we use an instrumental variable that isolates exogenous variation in out‐of‐pocket spending during pregnancy+delivery: the plan‐month of delivery. This approach estimates the local average treatment effect (LATE) of pregnancy+delivery out‐of‐pocket spending on postpartum utilization among birthing people whose pregnancy+delivery out‐of‐pocket costs are higher due to their plan‐month of delivery (the “compliers”).

Figure 1 shows how delivering early in the plan year leads not only to higher pregnancy+delivery out‐of‐pocket spending (which can lead to an income effect), but also to lower postpartum out‐of‐pocket spending (which can lead to a price effect); this mechanical relationship is due to the non‐linear cost‐sharing design of plans with deductibles, as previously described. Our LATE estimates therefore encompass both the income and price effect, among compliers. These two effects cannot be disentangled. We interpret the magnitude and direction of our LATE estimates as the combination of these price and income effects.

**FIGURE 2:**
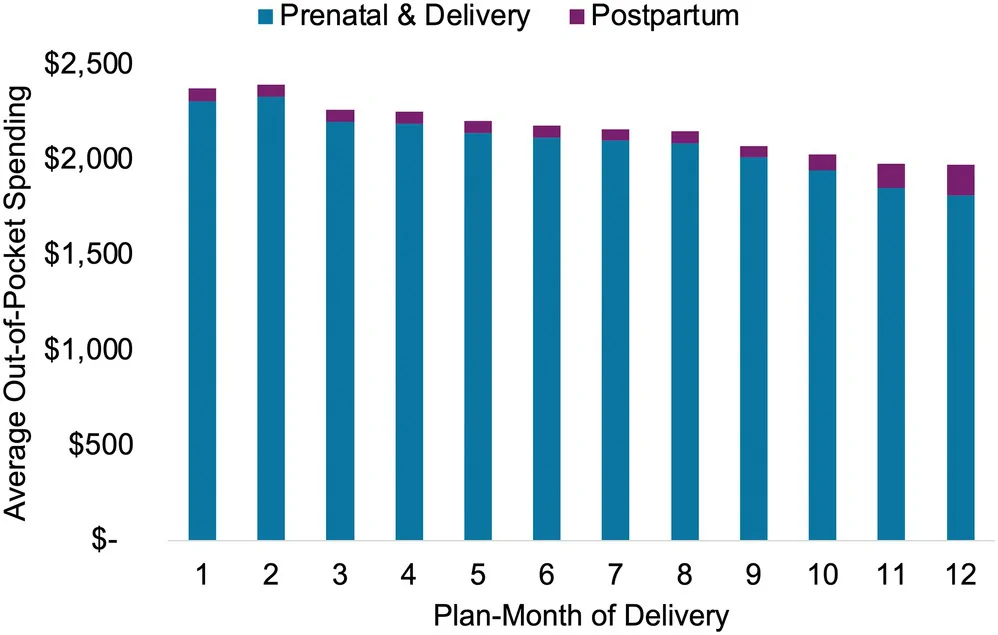
Out‐of‐pocket spending during the prenatal and delivery, and postpartum, periods, by plan‐month of delivery. *Note:* The figure shows average total out‐of‐pocket spending for the prenatal and delivery periods (blue) and 3 months postpartum (purple) among the full sample of maternity episodes (episodes with deliveries in plan‐months 1–12); characteristics of this sample are shown in Appendix D. Out‐of‐pocket spending disaggregated into prenatal spending and delivery spending by plan‐month of delivery is shown in Appendix G.

This approach and interpretation require four assumptions, all of which are conditional on the covariates that we include in all of our models. First, the plan‐month of delivery must be associated with pregnancy+delivery out‐of‐pocket spending. We examine this graphically (Figure 2) and report the F‐statistic from our first‐stage regressions. Importantly, all patients in our sample have a delivery, regardless of plan‐month of delivery. Second, the exclusion restriction requires that the plan‐month of delivery must only affect postpartum utilization through its effect on out‐of‐pocket spending. While this assumption is not empirically testable, we support it by showing minimal relationships between plan‐month of delivery and birthing people's observable characteristics (Appendix F) and with their total spending (Appendix G).

Third, the plan‐month of delivery must be as good as randomly assigned, which we support with evidence that births are evenly distributed across the plan‐year (Appendix D) and plan‐year start dates are distributed through the calendar year with the most frequent months January (58.9%), July (14.0%), August (5.5%), and September (4.1%) (Appendix H). Finally, we assume that the instrument's effects are monotonic, meaning that there are no “defiers,” or birthing people who have higher out‐of‐pocket pregnancy+delivery spending due to delivering late in their health plan year. This is supported by the mechanical relationship between nonlinear health plan contracts and out‐of‐pocket spending, and by prior literature showing that consumers' spending and use of health care services are highly responsive to this contract structure [15, 16, 17].

### 2.6 Statistical Analyses

We run two‐stage least squares linear regression models to estimate the LATEs. In our primary models, we use a dichotomized version of plan‐month of delivery that is equal to 0 for people delivering in plan‐months 1–3 and equal to 1 for those delivering in plan‐months 10–12; those delivering in plan‐months 4–9 are not included. This approach compares those whose delivery timing assigns them to the highest (plan‐months 1–3) or lowest (plan‐months 10–12) pregnancy+delivery out‐of‐pocket costs. We also show results with the continuous plan‐month of delivery instrument, including the full sample (Appendixes D and E).

All models are adjusted for the birthing person's age, race and ethnicity, product type (HMO vs. PPO), high‐deductible health plan enrollment, and include fixed effects for state and the calendar month–year of delivery to account for any seasonality or time trends (e.g., due to the COVID‐19 pandemic). We also estimate our two‐stage least squares models in the subgroup of birthing people who were enrolled in a high‐deductible health plan, as their out‐of‐pocket spending may be more strongly influenced by their plan‐month of delivery. As a robustness check, we estimate our models (1) including additional covariates that capture clinical features of the pregnancy and delivery: obstetric comorbidity index quartile, delivery episode length of stay (in tertiles) and delivery mode, and (2) for a sample with a high‐deductible of $2000 or higher.

We report our LATE estimates on three scales: (1) change per $100 difference in pregnancy+delivery out‐of‐pocket spending, (2) change per the approximate average difference in out‐of‐pocket spending between those delivering early and late in their health plan year, and (3) the percent change in the outcome (dividing the second scaled estimate by the mean outcome level among those delivering early in their health plan year). In the appendix, we show the results of the first stage adjusted regressions of pregnancy+delivery out‐of‐pocket spending on plan‐month of delivery (Appendix I). For comparison, we also show the results from reduced form regressions of each outcome on pregnancy+delivery out‐of‐pocket spending, which are subject to bias due to endogeneity between out‐of‐pocket spending and the clinical outcomes (Appendix J).

We used SAS, version 9.4, and R, version 4.5.1 for all analyses and considered two‐sided *p*‐values < 0.05 to indicate statistical significance. The (*Harvard Longwood Campus*) Institutional Review Board determined this study to be exempt.

## 3 Results

Our primary sample included 51,337 maternity episodes with deliveries in plan‐months 1–3 (49%) and 10–12 (51%) (Table 1). A majority of our sample lived in Massachusetts (61%), with 30% outside of New England. Most were ages 30–34 (44%) and were White non‐Hispanic (77%). Approximately one‐third (35%) of the sample were enrolled in a high‐deductible health plan, 63% were in a PPO, and 37% were in an HMO.

**TABLE 1:** Characteristics of maternity episodes.

	*N* Episodes	Percent of episodes
Number of birthing people	48,093	—
Pharmacy coverage	35,262	69%
Maternal age group (years)		
< 24 years old	3291	6%
25–29 years old	9298	18%
30–34 years old	22,412	44%
35–39 years old	13,468	26%
≥ 40 years old	2868	6%
Race/ethnicity		
Asian/Pacific Islander	5150	10%
Black	2240	4%
Hispanic	4380	9%
White	39,567	77%
State of residence, grouped		
Massachusetts	31,362	61%
Other New England	4798	9%
Non‐New England	15,177	30%
Delivery mode		
Vaginal delivery	34,714	68%
Cesarean delivery	16,623	32%
Comorbidities		
Hypertension	11,874	23.1%
Diabetes	9493	18.5%
Anxiety	13,048	25.4%
Depression	10,473	20.4%
Cardiac conditions	2211	4.3%
Substance use disorder	900	1.8%
Deductible level		
Low deductible (< $1500)	33,273	65%
High deductible (≥ $1500)	18,064	35%
Health plan type		
HMO	18,893	37%
PPO	32,444	63%
Delivery year		
2019	10,158	20%
2020	10,146	20%
2021	10,325	20%
2022	10,418	20%
2023	10,290	20%
Plan‐month of delivery (PMD)		
PMD 1–3	25,176	49%
PMD 10–12	26,161	51%

*Note:* Sample size *N* = 51,337; An individual could have more than one maternity episode in the sample over the study period 2019–2023; Results are shown for the primary analytic sample of maternity episodes with a delivery in plan‐months 1–3 or 10–12 (see Appendix D for characteristics of maternity episodes across the full plan year). Race and ethnicity are measured using a combination of self‐reported data and an imputation approach described in Gourevitch et al. [6]; We exclude race/ethnicity groups with very small samples (i.e., American Indian/Alaska Native, Native Hawaiian/Pacific Islander), or where we could not distinguish race/ethnicity within the group (i.e., other race, 2+ race/ethnicity).

Abbreviations: HMO, Health Maintenance Organization; PPO, Preferred Provider Organization.

Average out‐of‐pocket spending during pregnancy and delivery decreased monotonically with plan‐month of delivery, with those delivering in the first month of their plan year spending an average of $492, or 21%, more out‐of‐pocket than those delivering in the last month of their plan year ($2306 vs. $1814, Figure 2). Plan‐month of delivery also influenced postpartum spending: people who delivered in the last month of their health plan year and therefore faced a new deductible in the postpartum period spent $94, or 58%, more out‐of‐pocket on postpartum care compared to people who delivered in the first month of their plan year ($164 vs. $69). This pattern was even more pronounced for enrollees in high‐deductible plans, who faced a $918, or 25%, differential in pregnancy+delivery out‐of‐pocket spending between delivering in the first versus last plan‐month ($3660 vs. $2742, Appendix K). This supports the relevance assumption of our estimation approach: the instrument is strongly associated with the key exposure variable. This assumption is also supported by our two‐stage least squares models' F‐statistics, which were above 450 (Tables 2 and 3). Total spending, however, did not vary by plan‐month of delivery (Appendix K), nor were there meaningful differences between deliveries early versus late in the plan‐year (Appendix F), suggesting that the patterns observed in Figure 2 are driven by the cost‐sharing structure across the health plan year.

**TABLE 2:** Outcome means and instrumental variable estimates for primary analytic sample.

	Sample outcomes		2SLS LATE estimates		
	Mean	Std. dev.	Coef.	Std. err.	95% CI
Any outpatient visit	82.5%	0.4	0.53***	0.13	(0.28, 0.79)
Outpatient contact days	3.3	4.1	0.05***	0.01	(0.02, 0.07)
Outpatient visit for preventive/well care or postpartum evaluation	47.8%	0.5	0.41*	0.17	(0.08, 0.74)
Outpatient visit for contraceptive management	17.0%	0.4	0.19	0.13	(−0.06, 0.44)
Outpatient visit for other acute or chronic illness	69.9%	0.5	0.48**	0.16	(0.18, 0.79)
Outpatient visit for mental health	11.4%	0.3	0.25*	0.11	(0.04, 0.46)
Mental health prescription fill	16.1%	0.4	0.14	0.12	(−0.10, 0.39)
Any Emergency Department visit	6.1%	0.2	0.15^+^	0.08	(−0.01, 0.30)
F‐Statistic			475.4		
F‐Statistic, mental health prescription fill			524.2		

*Note:* 2SLS is two‐stage least squares; *N* = 51,337 except for mental health prescription fill outcome where *N* = 35,262. (a) *p*‐values correspond to ^+^ < 0.10, * < 0.05, ** < 0.01, *** < 0.001; (b) estimates are from 2SLS models with the following covariates: age, race and ethnicity, health plan type (HMO vs. PPO), high‐deductible health plan status, and fixed effects for state of residence and the calendar month–year of delivery; (c) estimates are scaled to represent changes per $100 of additional out‐of‐pocket spending; (d) the mental health prescription fill outcome has a different F‐Statistic because it is assessed only among those with linked pharmacy coverage (69% of the sample).

Abbreviation: LATE, local average treatment effect.

**TABLE 3:** Outcome means and instrumental variable estimates for high‐deductible sample.

	Sample outcomes		2SLS LATE estimates		
	Mean	Std. dev.	Coef.	Std. err.	95% CI
Any outpatient visit	81%	0.39	0.30***	0.09	(0.13, 0.48)
Outpatient contact days	3.22	4.08	0.02^+^	0.01	(−0.001, 0.04)
Outpatient visit for preventive/well care or postpartum evaluation	47%	0.50	0.22*	0.11	(0.005, 0.45)
Outpatient visit for contraceptive management	16%	0.37	0.05	0.08	(−0.12, 0.21)
Outpatient visit for other acute or chronic illness	68%	0.47	0.33**	0.11	(0.12, 0.54)
Outpatient visit for mental health	11%	0.31	0.12^+^	0.07	(−0.02, 0.26)
Mental health prescription fill	16%	0.36	0.04	0.09	(−0.13, 0.21)
Any Emergency Department visit	6%	0.24	−0.002	0.05	(−0.11, 0.1)
F‐Statistic			788.2		
F‐Statistic, mental health treatment			767.1		

*Note:* 2SLS is two‐stage least squares; High‐deductible plan has a deductible of $1500 or higher. *N* = 18,064 except for mental health prescription fill outcome where *N* = 14,023. (a) *p*‐values correspond to ^+^ < 0.10, * < 0.05, ** < 0.01, *** < 0.001; (b) estimates are from 2SLS models with the following covariates: age, race and ethnicity, health plan type (HMO vs. PPO), and fixed effects for state of residence and the calendar month–year of delivery; (c) estimates are scaled to represent changes per $100 of additional out‐of‐pocket spending; (d) the mental health treatment outcome has a different F‐Statistic because it is assessed only among those with linked pharmacy coverage (69% of the sample).

Abbreviation: LATE, local average treatment effect.

Use of postpartum outpatient care was common in the sample, with 82.5% having any outpatient visit and an average of 3.3 outpatient visit days following delivery (Table 2). Nearly half (47.8%) had an outpatient visit for preventive care or a well visit, 17.0% had a visit for contraceptive management, 69.9% had a visit for other acute or chronic care, 11.4% had an outpatient mental health visit, and 6.1% visited the emergency department. Among the subsample with BCBSMA pharmacy coverage, 16.1% filled a prescription for a mental health medication. Appendix L presents outpatient care use among only those delivering in plan‐months 1–3.

We find that having exogenously higher pregnancy+delivery out‐of‐pocket spending, which typically corresponds with lower cost‐sharing during the postpartum period, led to modest increases in postpartum outpatient care (Table 2). An additional $100 in pregnancy+delivery out‐of‐pocket spending led to a 0.53 percentage point (pp) increase in use of any outpatient care (95% CI [0.28, 0.79]); for the average observed differential in out‐of‐pocket spending between those who deliver early vs. late in the plan year (approximately $500), that amounts to a 2.7 percentage point, or 3.2%, increase in use of outpatient care (Appendix M). We also found that a $100 increase in pregnancy+delivery out‐of‐pocket spending led to more outpatient contact days (0.05 pp, 95% CI [0.02, 0.07]), outpatient visits for preventive/well care (0.41 pp, 95% CI [0.08, 0.74]), outpatient mental health visits (0.25 pp, 95% CI [0.04, 0.46]), and other outpatient visits (0.48 pp, 95% CI [0.18, 0.79]); increases in use of the emergency department were statistically significant at the 0.10 level (0.15 pp, 95% CI [−0.01, 0.30]). We found no statistically significant impacts on outpatient visits for contraceptive management (0.19 pp, 95% CI [−0.06, 0.44]) or prescription fills for mental health medications (0.23 pp, 95% CI [−0.05, 0.50]). Our results were consistent when covariates capturing clinical risk were included (Appendix O) and when the instrument was defined as the continuous plan‐month of delivery (Appendix E). These results are different from the (likely biased) reduced form estimates, which find no statistically significant effect of OOP spending on use of care except for mental health treatment (Appendix J).

In the subgroup with a deductible of $1500 or higher, the effects of pregnancy+delivery out‐of‐pocket spending on use of postpartum care were similar (Table 3). An additional $100 of pregnancy+delivery out‐of‐pocket spending led to a 0.30 percentage point increase in use of any postpartum outpatient care (95% CI [0.13, 0.48]); across the average differential in out‐of‐pocket spending between those who deliver at the beginning versus the end of their plan year (approximately $900), this amounts to a 2.7 percentage point, or 3.3%, increase in use of postpartum outpatient care (Appendix N). In this subgroup, we also found statistically significant increases between pregnancy+delivery out‐of‐pocket spending and visits for preventive/well care (0.22 pp, 95% CI [0.005, 0.45]), and other visits (0.33 pp, 95% CI [0.12, 0.54]). The results from a subgroup with higher deductibles of $2000 or more were similar in significance and direction, and the magnitude of the effects was slightly larger (Appendix P).

## 4 Discussion

Maternal health researchers, advocates, and policymakers are concerned about the high out‐of‐pocket costs for maternity care, but little work has examined how they impact use of care. In this study of commercially insured birthing people, we show that health plan benefit cycles expose people to different levels of pregnancy+delivery and postpartum cost‐sharing due to the timing of their delivery. People who deliver early in their plan year have higher pregnancy+delivery out‐of‐pocket spending and lower out‐of‐pocket spending during the first 3 months postpartum. We find that higher pregnancy+delivery costs due to delivering early in one's health plan year led to modest but clinically meaningful increases in use of postpartum care.

Our findings suggest that outpatient visits may be the most responsive to differences in out‐of‐pocket costs. Emergency department visits were only impacted at the 0.10 significance level in the full sample, though we also did not have as much power to detect changes in this infrequent outcome. We observed an increase in outpatient visits for mental health care in the full sample, but no impact on prescription fills for mental health medications. Responsiveness to cost‐sharing may differ for prescription drugs versus other medical care, though we are limited in our interpretation of this finding because use of mental health medications is estimated using a subsample (i.e., we only observe pharmacy coverage for two‐thirds of the study sample).

The estimates in this analysis encompass both an “income effect” (wherein patients respond to higher pregnancy+delivery bills by reducing postpartum utilization) and a “price effect” (wherein patients respond to lower cost‐sharing in the postpartum period—due to meeting their second deductible during their delivery—by using more care). Though we cannot disentangle these two mechanisms, our findings that higher pregnancy+delivery costs lead to higher postpartum utilization are consistent with the interpretation that the price effect dominates. This is different than our initial hypothesis, as we expected that the larger relative magnitude of pregnancy+delivery care bills would lead the income effect to dominate. In this setting, facing lower prices for postpartum care may have more influence on utilization than having received a large bill for pregnancy+delivery care [10]. This finding adds to the literature showing that consumers respond to healthcare cost‐sharing with myopia [15, 16, 17]. Additional research is needed to determine conclusively that this price effect dominates the income effect of a large expenditure in the maternity care context.

Increasing use of postpartum care has been a national priority given the high burdens of physical and mental health conditions during the recovery from childbirth [18, 19]. The role of out‐of‐pocket costs following delivery has been relatively absent from discussions of barriers to postpartum care, but these findings suggest that these costs are worthy of additional policy and research attention. State and federal policymakers are considering proposals to reduce out‐of‐pocket costs for maternity care in commercial insurance, which BCBSMA has supported [28]. Some states' proposals are focused on cost‐sharing during the pregnancy and delivery periods only [8, 9]. Our findings underscore the importance of including the postpartum period in the cost‐sharing exemption, as in the proposed federal legislation [8]. If only the pregnancy and delivery periods are exempt, many fewer birthing people would meet their deductibles during the delivery episode, leaving them exposed to higher cost‐sharing during the postpartum period. This could have the unintended consequence of reducing use of postpartum care. Relatedly, though routine prenatal and postpartum visits should be exempt from cost sharing under the Affordable Care Act, patients may not be aware of this exemption, and evidence from other settings shows that they may still face costs for these visits [29]. Thus, policies to reduce maternity and postpartum cost‐sharing may need to be accompanied by efforts to ensure that the policies are implemented as intended and to make patients aware of the cost‐sharing exemptions in order to increase postpartum care use.

While this study fills an important gap in the literature by rigorously identifying how out‐of‐pocket costs affect postpartum care utilization, it is not without limitations. First, we study only one health insurance carrier, and the out‐of‐pocket spending for birthing people in its plans is lower, on average, than other samples characterized in the literature [1]. Our data, however, allow us to observe detailed health plan characteristics including cost‐sharing structure and the exact month of the start of the health plan year; we find that less than 60% of enrollees' health plan years began in January (Appendix H), suggesting that assuming January start dates is a limitation in prior work [23]. Second, our results are interpreted as local average treatment effects and only apply to the “complier” population—individuals who had higher pregnancy+delivery out‐of‐pocket costs due to the timing of their delivery. The mechanical relationship between the health plan contract design and out‐of‐pocket costs suggests that enrollees should be compliers. Our results may not generalize to the impacts of having higher pregnancy+delivery out‐of‐pocket costs for other reasons, such as being higher risk, or delivering at a high‐price hospital. In addition, we require continuous enrollment from pregnancy through 3‐months postpartum for inclusion in the sample, and the results may not generalize to patients who experience insurance changes or churn. Finally, due to use of global billing codes that include one postpartum visit (i.e., exempt from cost‐sharing) in the payment for obstetric care, we may underestimate the level of routine postpartum care utilization in our sample. However, we expect any measurement error to be balanced across the values of both the treatment and instrumental variable, and therefore not a source of bias.

## 5 Conclusions

Benefit design and out‐of‐pocket costs during pregnancy+delivery care can influence use of postpartum care among commercially insured birthing people, and our results suggest that lower postpartum cost‐sharing can increase use of recommended outpatient care following childbirth. As health care providers and policymakers seek strategies for increasing use of postpartum care, it is important to consider the role of out‐of‐pocket costs. Policy proposals aiming to eliminate out‐of‐pocket costs for maternity care should also consider including the postpartum period. Limiting these costs may be an effective strategy for increasing postpartum care utilization and preventing adverse maternal outcomes following childbirth.

## Funding

This work was supported by the Patrick and Catherine Weldon Donaghue Medical Research Foundation.

## Conflicts of Interest

The authors declare no conflicts of interest.

## Supporting information


**Appendix A.** Sample distribution of days between delivery and provider payment date.
**Appendix B**. Billing codes used to identify delivery episodes.
**Appendix C**. Outcome definitions.
**Appendix D**. Characteristics of maternity episodes across plan‐months of delivery 1–12 (full sample, *N* = 104,388).
**Appendix E**. Outcome means and instrumental variable estimates for continuous plan‐month of delivery instrument.
**Appendix F**. Observable characteristics of maternity episodes by plan‐month of delivery.
**Appendix G**. Spending by plan‐month of delivery.
**Appendix H**. Full sample distribution of the calendar month of plan start date.
**Appendix I**. First stage regression results for binary instrumental variable.
**Appendix J**. Reduced form regression results.
**Appendix K**. High‐deductible sample out‐of‐pocket spending during the prenatal and delivery, and postpartum, periods, by plan‐month of delivery.
**Appendix L**. Sample means among those delivering in plan‐months 1–3, full and high‐deductible samples.
**Appendix M**. Scaling of local average treatment effects (LATEs) for primary analytic sample.
**Appendix N**. Scaling of local average treatment effects (LATEs) for high‐deductible sample.
**Appendix O**. Instrumental variable estimates, including clinical covariates.
**Appendix P**. Outcome means and instrumental variable estimates for higher deductible sample (Deductible $2000 or higher).

## Data Availability

Research data are subject to confidentiality protections and are not able to be shared.
