# Supplementary material for: Delivery Timing, Out‐Of‐Pocket Maternity Costs, and Postpartum Care Utilization: An Instrumental Variable Analysis Among Commercially Insured Women
Source: Health Serv Res. 2026 Sep 2;61(5):e70161. doi: 10.1111/1475-6773.70161 (PMC13535673; doi:10.1111/1475-6773.70161)
Supplement: Supplementary file 1 [file HESR-61-e70161-s001.docx]

**List of Appendices**

**Appendix A.** Sample Distribution of Days between Delivery and Provider Payment Date

**Appendix B.** Billing Codes Used to Identify Delivery Episodes

**Appendix C.** Outcome Definitions

**Appendix D.** Characteristics of Maternity Episodes Across Plan-Months of Delivery 1-12 (Full Sample, N=104,388)

**Appendix E**. Outcome Means and Instrumental Variable Estimates for Continuous Plan-Month of Delivery Instrument

**Appendix F.** Observable Characteristics of Maternity Episodes by Plan-Month of Delivery

**Appendix G.** Spending by Plan-Month of Delivery

**Appendix H.** Full Sample Distribution of the Calendar Month of Plan Start Date

**Appendix I.** First Stage Regression Results for Binary Instrumental Variable

**Appendix J.** Reduced Form Regression Results

**Appendix K.** High-Deductible Sample Out-of-Pocket Spending During the Prenatal & Delivery, and Postpartum, Periods, by Plan-Month of Delivery

**Appendix L.** Sample Means Among Those Delivering in Plan-Months 1-3, Full and High-Deductible Samples

**Appendix M.** Scaling of Local Average Treatment Effects (LATEs) for Primary Analytic Sample

**Appendix N.** Scaling of Local Average Treatment Effects (LATEs) for High-Deductible Sample

**Appendix O.** Instrumental Variable Estimates, Including Clinical Covariates

**Appendix P**. Outcome Means and Instrumental Variable Estimates for Higher Deductible sample (Deductible $2000 or higher)

**Appendix A.** Sample Distribution of Days between Delivery and Provider Payment Date

|  | Number of Days |
| --- | --- |
| Minimum | 5 |
| 25th Percentile | 18 |
| 50th Percentile | 22 |
| 75th Percentile | 28 |
| Average | 39 |

**Appendix B.** Billing Codes Used to Identify Delivery Episodes

| **Code Type** | **Code** | **Cesarean Section** | **Code Description** |
| --- | --- | --- | --- |
| CPT | 59510 | X | Routine obstetric care including antepartum care, cesarean delivery, and postpartum care |
| CPT | 59514 | X | Cesarean delivery only |
| CPT | 59515 | X | Cesarean delivery only; including postpartum care |
| CPT | 59618 | X | Routine obstetric care including antepartum care, cesarean delivery, and postpartum care, following attempted vaginal delivery after previous cesarean delivery |
| CPT | 59620 | X | Cesarean delivery only, following attempted vaginal delivery after previous cesarean delivery |
| CPT | 59622 | X | Cesarean delivery only, following attempted vaginal delivery after previous cesarean delivery; including postpartum care |
| CPT | 59400 |  | Routine obstetric care including antepartum care, vaginal delivery (with or without episiotomy, and/or forceps) and postpartum care |
| CPT | 59409 |  | Vaginal delivery only (with or without episiotomy and/or forceps); |
| CPT | 59410 |  | Vaginal delivery only (with or without episiotomy and/or forceps); including postpartum care |
| CPT | 59610 |  | Routine obstetric care including antepartum care, vaginal delivery (with or without episiotomy, and/or forceps) and postpartum care, after previous cesarean delivery |
| CPT | 59612 |  | Vaginal delivery only, after previous cesarean delivery (with or without episiotomy and/or forceps) |
| CPT | 59614 |  | Vaginal delivery only, after previous cesarean delivery (with or without episiotomy and/or forceps); including postpartum care |
| DRG | 540 | X | Cesarean Delivery |
| DRG | 541 |  | Vaginal Delivery W Sterilization &/Or D&C |
| DRG | 541 |  | Vaginal Delivery W/ Sterilization &/Or D&C |
| DRG | 542 |  | Vaginal Delivery W/ Complicating Procedures Exc Sterilization &/Or D&C |
| DRG | 542 |  | Vaginal Delivery W/ Complicating Procedures Exc St |
| DRG | 560 |  | Vaginal Delivery |
| DRG | 766 | X | Cesarean Section Without Cc/Mcc |
| DRG | 768 |  | Vaginal Delivery W O.R. Proc Except Steril &/Or D&C |
| DRG | 774 |  | Vaginal Delivery With Complicating Diagnoses |
| DRG | 775 |  | Vaginal Delivery W/O Complicating Diagnoses |
| DRG | 775 |  | Vaginal Delivery Without Complicating Diagnoses |
| ICD10 Proc | 10D00Z0 |  | Extraction of Products of Conception High Open Approach |
| ICD10 Proc | 10D00Z1 |  | Extraction of Products of Conception Low Open Approach |
| ICD10 Proc | 10D00Z2 |  | Extraction of Products of Conception Extraperitoneal Open Approach |
| ICD10 Proc | 10D07Z3 |  | Extraction of Products of Conception Low Forceps Via Opening |
| ICD10 Proc | 10D07Z4 |  | Extraction of Products of Conception Mid Forceps Via Opening |
| ICD10 Proc | 10D07Z5 |  | Extraction of Products of Conception High Forceps Via Opening |
| ICD10 Proc | 10D07Z6 |  | Extraction of Products of Conception Vacuum Via Opening |
| ICD10 Proc | 10D07Z7 |  | Extraction of Products of Conception Int Version Via Opening |
| ICD10 Proc | 10D07Z8 |  | Extraction of Products of Conception Other Via Opening |
| ICD10 Proc | 10E0XZZ |  | Delivery of Products of Conception External Approach |

**Appendix C.** Outcome Definitions

| **Outpatient Care** | |
| --- | --- |
| Any outpatient visit | Any outpatient visit, excluding emergency department visits |
| Number of outpatient contact days | Number of days with any outpatient visit (as defined above) |
| Outpatient visit for preventive/well care or postpartum evaluation | Any outpatient visit with any of the following billing codes   - CPTs for preventive care: 99384-99387 99394-99397 99381 99382 99383 99391 99392 99393 99401 99402 99403 99404 99411 99412 99429 (Smith et al. 2025) - ICD-10 diagnosis for general exam: Z00.00, Z00.01, Z00.121, Z00.129 (Smith et al. 2025), Z01.411, Z01.419 (Steenland et al. 2021) - ICD-10 diagnosis for postpartum evaluation: Z39* (Smith et al. 2025) - CPT code for postpartum evaluation: 59430, 0503F |
| Outpatient visit for contraceptive management | Any outpatient visit with a diagnosis code for contraceptive management: Z30* |
| Outpatient visit for other acute or chronic illness | Any other outpatient visit not captured in the two above categories (Smith et al. 2025) |
| **Other Care** | |
| Mental health treatment (only among patients with pharmacy claims) | Either of the following   - A medical claim with one of the following CPT codes for outpatient mental health care: 90804-90815; 90816-90819; 90820-90824; 90826-90829; 90832-90834; 90836- 90840; 90841-90844; 90845-90847; 90849; 90853; 90855; 90857; 90862; 90875; 90876 (Gordon et al. 2024) - A pharmacy claim for a prescription fill for an antidepressant or anxiolytic medication (Rokicki et al. 2022) |
| Emergency Department Visit | Any ER visit (defined using ED place of service field or CPT code for emergency department services (99281-99285) |

**Appendix D.** Characteristics of Maternity Episodes Across Plan-Months of Delivery 1-12 (Full Sample, N=104,388)

|  | N Maternity Episodes | Percent of Maternity Episodes |
| --- | --- | --- |
| Number of Birthing People^a^ | 91220 |  |
| Pharmacy Coverage | 70676 | 68% |
| Race/Ethnicity |  |  |
| Asian/Pacific Islander | 10567 | 10% |
| Black | 4503 | 4% |
| Hispanic | 8799 | 8% |
| White | 80519 | 77% |
| Maternal Age Group (Years) |  |  |
| < 24 Years Old | 6505 | 6% |
| 25 -29 Years Old | 9298 | 18% |
| 30 - 34 Years Old | 22412 | 44% |
| 35 - 39 Years Old | 13468 | 26% |
| ≥ 40 Years Old | 5636 | 5% |
| State of Residence, Grouped |  |  |
| Massachusetts | 63623 | 61% |
| Other New England | 9765 | 9% |
| Non-New England | 31000 | 30% |
| Delivery Mode |  |  |
| Vaginal Delivery | 57343 | 63% |
| Cesarean Delivery | 33877 | 33% |
| Deductible Level |  |  |
| Low Deductible (< $1,500) | 67537 | 65% |
| High Deductible (≥ $1,500) | 36851 | 35% |
| Health Plan Type |  |  |
| Health Maintenance Organization (HMO) | 37671 | 36% |
| Preferred Provider Organization (PPO) | 66717 | 64% |
| Delivery Year |  |  |
| 2019 | 20714 | 20% |
| 2020 | 20479 | 20% |
| 2021 | 21160 | 20% |
| 2022 | 21209 | 20% |
| 2023 | 20826 | 20% |
| Plan-Month of Delivery (PMD) |  |  |
| 1 | 8332 | 8% |
| 2 | 8159 | 8% |
| 3 | 8685 | 8% |
| 4 | 8663 | 8% |
| 5 | 8866 | 8% |
| 6 | 8616 | 8% |
| 7 | 9060 | 9% |
| 8 | 8992 | 9% |
| 9 | 8854 | 8% |
| 10 | 8939 | 9% |
| 11 | 8820 | 8% |
| 12 | 8402 | 8% |
| Notes: (a) Individuals could have more than one maternity episode in the sample over the study period | | |

**Appendix E**. Outcome Means and Instrumental Variable Estimates for Continuous Plan-Month of Delivery Instrument

|  | **Sample Outcomes** | | **2SLS LATE**  **Estimates** | | |
| --- | --- | --- | --- | --- | --- |
|  | Mean | Std. Dev | Coef. | Std. Err | 95% CI |
| Any outpatient visit | 82.5% | 0.38 | 0.33** | 0.10 | (0.13, 0.53) |
| Outpatient contact days | 3.29 | 4.06 | 0.03** | 0.01 | (0.013, 0.06) |
| Outpatient visit for preventive/well care or postpartum evaluation | 47.8% | 0.5 | 0.42** | 0.13 | (0.156, 0.68) |
| Outpatient visit for contraceptive management | 17.0% | 0.376 | 0.07 | 0.10 | (-0.12, 0.27) |
| Outpatient visit for other acute or chronic illness | 69.9% | 0.459 | 0.33** | 0.12 | (0.08, 0.57) |
| Outpatient visit for mental health | 11.4% | 0.3 | 0.14^+^ | 0.08 | (-0.03, 0.30) |
| Mental health prescription fill | 16.1% | 0.4 | 0.13 | 0.10 | (-0.08, 0.33) |
| Any ED visit | 6.1% | 0.239 | 0.10 | 0.06 | (-0.02, 0.21) |
| F-Statistic | | | 69.9 | | |
| F-Statistic, mental health prescription | | | 70.0 | | |
| *Notes:* N=104,388 (a) p-values correspond to ^+^<0.10, * < 0.05, ** < 0.01, *** < 0.001; (b) estimates are from two-stage least squares models (2SLS) with the following covariates: age, race and ethnicity, health plan type (HMO vs. PPO), high-deductible health plan status, and fixed effects for state of residence and the calendar month-year of delivery; (c) estimates are scaled to represent changes per $100 of additional out-of-pocket spending; (d) the mental health prescription outcome has a different F-Statistic because it is assessed only among those with linked pharmacy coverage (69% of the sample). | | | | | |

**Appendix F.** Observable Characteristics of Maternity Episodes by Plan-Month of Delivery

|  | **PMD 1-3** | | **PMD 10-12** | | **χ^2^ p-value** | | **Cramer’s V** |
| --- | --- | --- | --- | --- | --- | --- | --- |
|  | N | % | N | % | |  |  |
| Race/Ethnicity |  |  |  |  | | 0.5044 | 0.0068 |
| Asian/Pacific Islander | 2479 | 10% | 2671 | 10% | |  |  |
| Black | 1085 | 4% | 1155 | 4% | |  |  |
| Hispanic | 2153 | 9% | 2227 | 9% | |  |  |
| White | 19459 | 77% | 20108 | 77% | |  |  |
| Maternal Age Group (Years) |  |  |  |  | | 0.4011 | 0.0110 |
| < 20 Years Old | 163 | 1% | 205 | 1% | |  |  |
| 20 - 24 Years Old | 1465 | 6% | 1458 | 6% | |  |  |
| 25 -29 Years Old | 4582 | 18% | 4716 | 18% | |  |  |
| 30 - 34 Years Old | 10982 | 44% | 11430 | 44% | |  |  |
| 35 - 39 Years Old | 6593 | 26% | 6875 | 26% | |  |  |
| 40 - 44 Years Old | 1301 | 5% | 1368 | 5% | |  |  |
| > 44 Years Old | 90 | 0% | 109 | 0% | |  |  |
| Leonard Comorbidity Score Quartile |  |  |  |  | |  |  |
| Q1 (lowest risk) | 6879 | 27% | 6934 | 27% | | 0.0376 | 0.0128 |
| Q2 | 5855 | 23% | 6201 | 24% | |  |  |
| Q3 | 6626 | 26% | 6778 | 26% | |  |  |
| Q4 (highest risk) | 5816 | 23% | 6248 | 24% | |  |  |
| Geographic Location |  |  |  |  | | 0.0037 | 0.0148 |
| Massachusetts | 15294 | 61% | 16068 | 61% | |  |  |
| Other New England | 2463 | 10% | 2335 | 9% | |  |  |
| Not New England | 7419 | 29% | 7758 | 30% | |  |  |
| Deductible Level |  |  |  |  | | 0.3879 | 0.0038 |
| Low Deductible (< $1,500) | 16364 | 65% | 16909 | 65% | |  |  |
| High Deductible (≥ $1,500) | 8812 | 35% | 9252 | 35% | |  |  |
| Health Plan Type |  |  |  |  | | 0.189 | 0.0058 |
| HMO | 9937 | 37% | 9556 | 37% | |  |  |
| PPO | 15839 | 63% | 16605 | 63% | |  |  |
| Year of Delivery |  |  |  |  | | 0.0339 | 0.0142 |
| 2019 | 4852 | 19% | 5306 | 20% | |  |  |
| 2020 | 5043 | 20% | 5103 | 20% | |  |  |
| 2021 | 5045 | 20% | 5280 | 20% | |  |  |
| 2022 | 5121 | 20% | 5297 | 20% | |  |  |
| 2023 | 5115 | 20% | 5175 | 20% | |  |  |

Notes: PMD is plan-month of delivery; HMO is Health Maintenance Organization; PPO is Preferred Provider Organization. N= N=51,337. Cramer’s V value of less than 0.1 indicates a weak association (i.e., no difference in the distribution of PMD 1-3 vs PMD 10-12).^1^

**Appendix G.** Spending by Plan-Month of Delivery

**Panel A:** Average Total Pregnancy + Delivery Costs by Plan-Month of Delivery

**Panel B:** Average Out-of-Pocket (OOP) Prenatal vs Delivery Costs, by Plan-Month of Delivery

| Plan-Month of Delivery | Average prenatal OOP spending | Average delivery  OOP spending |
| --- | --- | --- |
| 1 | $463 | $1,850 |
| 2 | $515 | $1,827 |
| 3 | $506 | $ 1,695 |
| 4 | $509 | $1,692 |
| 5 | $525 | $1,622 |
| 6 | $528 | $1,600 |
| 7 | $537 | $1,572 |
| 8 | $570 | $1,522 |
| 9 | $569 | $1,450 |
| 10 | $572 | $1,398 |
| 11 | $536 | $1,369 |
| 12 | $507 | $1,369 |

Notes: N=104,388

**Appendix H.** Full Sample Distribution of the Calendar Month of Plan Start Date

| Plan Year Start | N Deliveries | Percent of Deliveries |
| --- | --- | --- |
| January | 61464 | 58.9% |
| February | 1258 | 1.2% |
| March | 2599 | 2.5% |
| April | 2743 | 2.6% |
| May | 2615 | 2.5% |
| June | 2932 | 2.8% |
| July | 14649 | 14.0% |
| August | 5720 | 5.5% |
| September | 4326 | 4.1% |
| October | 1735 | 1.7% |
| November | 1433 | 1.4% |
| December | 2914 | 2.8% |
|  |  |  |

Notes: N=104,388

**Appendix I.** First Stage Regression Results for Binary Instrumental Variable

|  | **Coef. ($)** | **Std. Error** | **P-Value** | **95% CI ($)** |
| --- | --- | --- | --- | --- |
| Primary Analytic Sample | 372.89 | 17.10 | <0.001 | (339.37, 406.42) |
| Pharmacy Sample | 388.80 | 17.0 | <0.001 | (355.52, 422.09) |
| High-Deductible Plan Sample | 839.91 | 29.92 | <0.001 | (781.26, 898.55) |
| Notes: Primary analytic sample N=51,337, Pharmacy sample N=35,262, High-deductible sample N=18,064. Results are from a linear regression of out-of-pocket costs on the binary plan-month of delivery indicator, fully adjusted for covariates used in the IV estimation. | | | | |

**Appendix J.** Reduced Form Regression Results

|  | **Coef.** | **Std. Err** | **95% CI** |
| --- | --- | --- | --- |
| Any outpatient visit | -0.02 | 0.01 | (-0.04, 0.01) |
| Outpatient contact days | 0.003* | 0.001 | (0.001, 0.01) |
| Outpatient visit for preventive/well care or postpartum evaluation | -0.03 | 0.02 | (-0.06, 0.00) |
| Outpatient visit for contraceptive management | 0.00 | 0.01 | (-0.03, 0.02) |
| Outpatient visit for other acute or chronic illness | -0.01 | 0.01 | (-0.04, 0.02) |
| Outpatient visit for mental health | 0.06*** | 0.01 | (0.04, 0.08) |
| Mental health prescription fill | 0.07*** | 0.02 | (0.04, 0.10) |
| Any Emergency Department visit | 0.001 | 0.01 | (-0.01, 0.02) |
| *Notes:* Includes primary analytic sample, N=51,337, for all outcomes except for mental health prescription fill outcome (N=35,262). (a) p-values correspond to * < 0.05, ** < 0.01, *** < 0.00; (b) linear regression of outcome on out-of-pocket spending, adjusted for age, race and ethnicity, health plan type (HMO vs. PPO), high-deductible health plan status, and fixed effects for state of residence and the calendar month-year of delivery (i.e., the covariates used in the instrumental variable estimation); (c) estimates are scaled to represent changes per $100 of additional out-of-pocket spending; (d) the mental health prescription outcome is assessed only among those with linked pharmacy coverage (69% of the sample) | | | |

**Appendix K.** High-Deductible Sample Out-of-Pocket Spending During the Prenatal & Delivery, and Postpartum, Periods, by Plan-Month of Delivery

Notes: OOP is out-of-pocket costs. High-deductible sample includes deliveries in health plans with a deductible of $1500 or greater. N=18,064

**Appendix L.** Sample Means Among Those Delivering in Plan-Months 1-3, Full and High-Deductible Samples

|  | **Primary Sample** | | **High-Deductible Subgroup** | |
| --- | --- | --- | --- | --- |
|  | Mean | Std. Dev | Mean | Std. Dev |
| Any outpatient visit | 82.4% | 0.4 | 82.3% | 0.4 |
| Outpatient contact days | 3.3 | 4.1 | 3.3 | 4.2 |
| Outpatient visit for preventive/well care or postpartum evaluation | 47.7% | 0.5 | 47.4% | 0.5 |
| Outpatient visit for contraceptive management | 17.0% | 0.4 | 16.5% | 0.4 |
| Outpatient visit for other acute or chronic illness | 69.8% | 0.5 | 69.2% | 0.5 |
| Outpatient visit for mental health | 11.4% | 0.3 | 11.3% | 0.3 |
| Mental health prescription fill | 16.3% | 0.4 | 16.1% | 0.4 |
| Any Emergency Department visit | 6.0% | 0.2 | 5.9% | 0.2 |
| *Note:* N=25,176; Outcome means for the full samples are shown in Table 2 for the primary sample and Table 3 for the high-deductible subgroup. High-deductible sample includes deliveries in health plans with a deductible of $1,500 or greater. | | | | |

**Appendix M.** Scaling of Local Average Treatment Effects (LATEs) for Primary Analytic Sample

|  | **(1) LATE per $100** of pregnancy+delivery OOP spending  (Table 2) | **(2) LATE scaled to approximate average difference in pregnancy+delivery** **OOP spending** between PMD 1-3 deliveries and PMD 10-12 deliveries ($500 for full sample) | **(3) Percent Change**: Scaled LATE (column 2) divided by mean outcome among PMD 1-3 deliveries (Appendix L) |  |
| --- | --- | --- | --- | --- |
| Any outpatient visit | 0.53 | 2.65 | 3.2% |  |
| Outpatient contact days | 0.05 | 0.25 | 0.1% |  |
| Outpatient visit for preventive/well care or postpartum evaluation | 0.41 | 2.05 | 4.3% |  |
| Outpatient visit for contraceptive management | 0.19 | 0.95 | 5.6% |  |
| Outpatient visit for other acute or chronic illness | 0.48 | 2.4 | 3.4% |  |
| Outpatient visit for mental health | 0.25 | 1.25 | 11.0% |  |
| Mental health prescription fill | 0.14 | 0.7 | 4.3% |  |
| Any ED visit | 0.15 | 0.75 | 12.5% |  |
| *Note:* OOP is Out-of-Pocket; PMD is Plan-Month of Delivery; ED is Emergency Department. N=51,337 for all outcomes except for mental health prescription fill outcome (N=35,262). Table 2 shows the full results corresponding to column 1, scaled to represent changes per $100 in pregnancy+delivery out-of-pocket spending. | | | | |

**Appendix N.** Scaling of Local Average Treatment Effects (LATEs) for High-Deductible Sample

|  | **(1) LATE per $100** of pregnancy+delivery OOP spending (Table 3) | **(2) LATE scaled to approximate average difference in pregnancy+delivery OOP spending** between PMD 1-3 deliveries and PMD 10-12 deliveries ($900 for full sample) | **(3) Percent Change**: Scaled LATE (column 2) divided by mean outcome among PMD 1-3 deliveries (Appendix L) |
| --- | --- | --- | --- |
| Any outpatient visit | 0.3 | 2.7 | 3.3% |
| Outpatient contact days | 0.02 | 0.18 | 0.1% |
| Outpatient visit for preventive/well care or postpartum evaluation | 0.22 | 1.98 | 4.2% |
| Outpatient visit for contraceptive management | 0.05 | 0.45 | 2.7% |
| Outpatient visit for other acute or chronic illness | 0.33 | 2.97 | 4.3% |
| Outpatient visit for mental health | 0.12 | 1.08 | 9.8% |
| Mental health prescription fill | 0.04 | 0.36 | 2.3% |
| Any ED visit | -0.002 | -0.018 | -0.3% |
| *Note:* OOP is Out-of-Pocket; PMD is Plan Month of Delivery; ED is Emergency Department. High-deductible sample includes deliveries in health plans with a deductible of $1,500 or greater. N=18,064 for all outcomes except for mental health prescription fill outcome (N=14,023). Table 3 shows the full results corresponding to column 1, scaled to represent changes per $100 in pregnancy+delivery out-of-pocket spending. | | | |

**Appendix O.** Instrumental Variable Estimates, Including Clinical Covariates

|  | **Primary Sample** | | | **HDHP Sample** | | |
| --- | --- | --- | --- | --- | --- | --- |
|  | Coef. | Std. Err | 95% CI | Coef. | Std. Err | 95% CI |
| Any outpatient visit | 0.53*** | 0.13 | (0.28, 0.79) | 0.31*** | 0.09 | (0.14, 0.48) |
| Outpatient contact days | 0.05*** | 0.01 | (0.02, 0.07) | 0.02* | 0.01 | (0.001, 0.04) |
| Outpatient visit for preventive/well care or postpartum evaluation | 0.41* | 0.17 | (0.08, 0.73) | 0.23* | 0.11 | (0.008, 0.45) |
| Outpatient visit for contraceptive management | 0.2 | 0.13 | (-0.04, 0.45) | 0.05 | 0.08 | (-0.11, 0.22) |
| Outpatient visit for other acute or chronic illness | 0.49** | 0.15 | (0.18, 0.79) | 0.34** | 0.11 | (0.14, 0.55) |
| Outpatient visit for mental health | 0.25^+^ | 0.11 | (0.04, 0.46) | 0.12 | 0.07 | (-0.01, 0.26) |
| Mental health prescription fill | 0.16 | 0.12 | (-0.08, 0.40) | 0.05 | 0.09 | (-0.12, 0.22) |
| Any ED visit | 0.15^+^ | 0.08 | (-0.01, 0.3) | -0.001 | 0.05 | (-0.1, 0.1) |
| F-Statistic | 475.4 | | | 788.1 | | |
| F-Statistic, mental health treatment | 523.8 | | | 767.1 | | |
| *Notes:* HDHP is high-deductible health plan; ED is Emergency Department. High-deductible sample includes deliveries in health plans with a deductible of $1,500 or greater. N=51,337 in primary sample and N=18,064 in high-deductible sample for all outcomes except for mental health prescription fill outcome (N=35,262 and N=14,023 respectively). (a) p-values correspond to ^+^<0.10, * < 0.05, ** < 0.01, *** < 0.001; (b) estimates are from two-stage least squares models (2SLS) with additional covariates for clinical risk (Leonard comorbidity index quartile, delivery length of stay, delivery mode) along with covariates included in the main models (age, race and ethnicity, health plan type (HMO vs. PPO), high-deductible health plan status (in the primary sample model, only), and fixed effects for state of residence and the calendar month-year of delivery); (c) estimates are scaled to represent changes per $100 of additional out-of-pocket spending; (d) the mental health treatment outcome has a different F-Statistic because it is assessed only among those with linked pharmacy coverage (69% of the sample) | | | | | | |

**Appendix P**. Outcome Means and Instrumental Variable Estimates for Higher Deductible sample (Deductible $2000 or higher)

|  | **Sample Outcomes** | | **2SLS LATE**  **Estimates** | | |
| --- | --- | --- | --- | --- | --- |
|  | Mean | Std. Dev | Coef. | Std. Err | 95% CI |
| Any outpatient visit | 81.8% | 38.5% | 0.37*** | 0.10 | (0.17, 0.57) |
| Outpatient contact days | 3.26 | 4.11 | 0.02* | 1.06 | (.0007, 0.04) |
| Outpatient visit for preventive/well care or postpartum evaluation | 47.2% | 49.9% | 0.23^+^ | 0.13 | (-0.03, 0.48) |
| Outpatient visit for contraceptive management | 16.8% | 37.4% | 0.06 | 0.10 | (-0.13, 0.25) |
| Outpatient visit for other acute or chronic illness | 69.1% | 46.2% | 0.41*** | 0.12 | (0.17 ,0.64) |
| Outpatient visit for mental health | 11.6% | 32.1% | 0.12 | 0.08 | (-04, 0.29) |
| Mental health prescription fill | 16.1% | 36.7% | 0.02 | 0.10 | (-0.17 , 0.22) |
| Any Emergency Department visit | 5.8% | 23.4% | -0.02 | 0.06 | (-0.13, 0.10) |
| F-Statistic | | |  | | |
| F-Statistic, mental health treatment | | |  | | |
| *Notes:* 2SLS is two-stage least squares; LATE is Local Average Treatment Effect; High-deductible plan has a deductible of $2000 or higher (N=12,169). (a) p-values correspond to ^+^<0.10, * < 0.05, ** < 0.01, *** < 0.001; (b) estimates are from two-stage least squares models (2SLS) with the following covariates: age, race and ethnicity, health plan type (HMO vs. PPO), and fixed effects for state of residence and the calendar month-year of delivery; (c) estimates are scaled to represent changes per $100 of additional out-of-pocket spending; (d) the mental health prescription outcome has a different F-Statistic because it is assessed only among those with linked pharmacy coverage (69% of the sample) | | | | | |

References

1.	Sapra RL, Saluja S. Understanding statistical association and correlation. *Current Medicine Research and Practice*. 2021;11(1):31-38. doi:10.4103/cmrp.cmrp_62_20
